# The impact of specific fracture characteristics of low-energy fractures of the pelvis on mortality

**DOI:** 10.1186/s12877-022-03223-z

**Published:** 2022-08-15

**Authors:** Michaela Ramser, Werner Vach, Nathalie Strub, Dieter Cadosch, Franziska Saxer, Henrik Eckardt

**Affiliations:** 1 Department of Orthopaedic and Traumatology Surgery, University Hospital Basel, 4031 Basel, Switzerland; 2 University of Basel, Basel, Switzerland; 3 Basel Academy for Quality and Research in Medicine, Basel, Switzerland; 4 Novartis Institutes for Biomedical Research, Novartis Campus, Basel, Switzerland

**Keywords:** Pelvic fracture, Frailty, Older patients, Mortality, Fracture characteristics, Fragility fractures of the pelvis, FFP

## Abstract

**Background:**

Pelvic fractures in older patients are associated with relevant morbidity and mortality. Both might be determined by fracture morphology and/or patient characteristics. The aim of this project is to investigate the prognostic value of specific fracture characteristics with respect to overall survival and to compare it with an established classification system.

**Methods:**

Retrospective analysis of patients ≥ 60 years, treated conservatively for a CT-scan verified, low-energy pelvic ring fracture between August 2006 and December 2018. Survival data was available from patients’ charts and cantonal or national registries.

The prognostic value of fracture characteristic describing the anterior and posterior involvement of the pelvic ring was investigated. This analysis was repeated after patients were stratified into a high-risk vs a low-risk group according to patient characteristic (age, gender, comorbidities, mobility, living situation). This allowed to assess the impact of the different fracture morphologies on mortality in fit vs. frail senior patients separately.

**Results:**

Overall, 428 patients (83.4% female) with a mean age of 83.7 years were included. Two thirds of patients were still living in their home and mobile without walking aid at baseline. In-hospital mortality was 0.7%, overall, one-year mortality 16.9%. An independent and significant association of age, gender and comorbidities to overall survival was found. Further, the occurrence of a horizontal sacral fracture as well as a ventral comminution or dislocation was associated with an increased mortality. The effect of a horizontal sacral fracture was more accentuated in low-risk patients while the ventral fracture components showed a larger effect on survival in high-risk patients.

**Conclusion:**

Specific fracture characteristics may indicate a higher risk of mortality in conservatively treated patients with a low-energy pelvic ring fracture. Hence, they should be taken into account in future treatment algorithms and decisions on patient management.

## Introduction

As the general population becomes older, the incidence of low-energy pelvic ring fractures increases [1]. Importantly, high mortality rates after these fractures have been reported [2]. One-year mortality ranges from 11–27% [3–11]. While the prognostic impact of hip fractures in older patients is widely accepted, the risk and excess mortality associated with pelvic fragility fractures is still underestimated and receives considerably less attention [12–14]. This becomes even more apparent from the continuing debate on the appropriate diagnostic tools, classification systems and treatment strategies [2, 15–19].

So far, age has been found to be a prognostic factor for mortality in patients with low-energy pelvic ring fractures [3]. Further, the AO-classification of pelvic fractures has been shown to correlate with mortality [20]. A correlation of the “fragility fractures of the pelvis” (FFP) classification to mortality was not found [21].

Here, we focus on single fracture characteristics of low-energy pelvic ring fractures which are also reflected in the FFP classification like the extent of the dorsal and ventral aspect of the pelvic ring fracture (absent, unilateral, bilateral), the presence/absence of a horizontal sacral fracture and the presence/absence of comminution and/or dislocation of the ventral fracture.

The aim of the current analysis is to evaluate these fracture characteristics considering their association with mortality and to assess prognostic factors in older patients with a low-energy pelvic ring fractures regarding outcome.

## Materials and methods

For the present analysis, all patients ≥ 60 years old treated for a CT scan verified low-energy pelvic ring fracture at our hospital between August 2006 and December 2018 were included unless a dissent for the use of routine data was documented. The study was approved by the competent ethical committee “Ethikkommission Nordwest- und Zentralschweiz; EKNZ” (Ref. 2017–01,859, ClinicalTrials.gov Identifier: NCT03476824). All methods were performed in accordance with the relevant guidelines and regulation. It followed applicable law as well as good clinical practice (GCP) and the Declaration of Helsinki.

Patients were identified by screening the electronic patients’ charts for the following keywords: pelvic ring fracture, pelvic fracture, ala fracture, sacrum fracture and ramus pubis fracture. We also screened for acetabular fractures in order not to miss fractures of the anterior wall as extension of a pubic ramus fracture. The injury mechanism from anamnestic information allowed a discrimination between patients with low- or high-energy injuries according to a predetermined list of possible trauma mechanisms. Patients with high-energy trauma like traffic accidents, bicycle accidents, falls on stairways or falls from considerable height other than standing position were excluded.

Additionally, the following six patient characteristics were collected from patients’ charts: age, gender, comorbidities (assessed by the Elixhauser Comorbidity Index (ECI) [22]), mobility in daily activities (assessed by the Parker Mobility score (PMS) [23], living situation, use of walking aids. Information on treatment (surgery vs. conservative) was obtained from the medical records. For the purpose of this analysis, only conservatively treated patients were considered.

Each CT scan was classified according to the traditional FFP classification [24] and the fracture additionally broken down into the description of five specific fracture characteristic:

- extent of the dorsal aspect of the pelvic ring fracture (absent, unilateral, bilateral)
- presence/absence of a horizontal sacral fracture
- extent of the ventral aspect of the pelvic ring fracture (absent, unilateral, bilateral)
- presence/absence of comminution of the ventral fracture
- presence/absence of a dislocation of the ventral fracture

Information on survival was obtained from patient charts as well as by contacting the registration office of the canton and the national pension scheme.

Survival times are defined as the time from first presentation and fracture diagnosis until death. If information on survival was obtained from the registration office of the canton, relocation to another canton as well as the end of the observation period (January 2020) were regarded as censoring events. If survival information was obtained from the national pension scheme, only the end of the observation period was regarded as censoring event.

Stratified Kaplan-Maier-curves are used to visualize the association of overall survival with single variables. Age, the PMS and the ECI were categorized for these analyses. The log rank test was used to assess the statistical significance of the differences between the strata shown.

The Cox proportional hazard model was used for multivariate analyses. Hazard ratios together with 95%-confidence intervals and *p-*values are reported. The extent of dorsal and ventral fractures was handled as continuous covariates.

For the development of a prognostic index, age, PMS and comorbidity enter the models as quadratic functions. Living status was handled as a categorical covariate. Missing values in the PMS, living status or use of walking aids were handled by the missing indicator approach, as these missing values are likely to be informative and carry prognostic information.

The independent prognostic value of the five fracture characteristics was assessed using a multivariable Cox model with all five fracture characteristics.

**Fig. 1:**
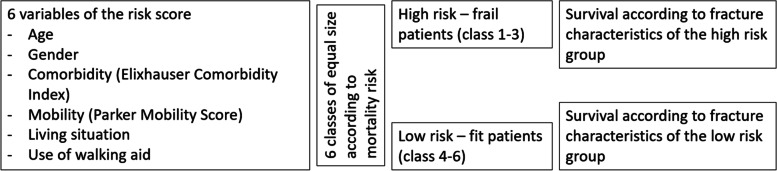
Variables of the risk score

The analyses on the correlation of specific fracture characteristics to mortality are then repeated separately in low-risk and high-risk patients using patient characteristics (age, gender, comorbidity, mobility, living situation and use of walking aid) for risk stratification (Fig. 1). Considering the prognostic value of the above-described patient characteristics, a stratification into six risk classes of equal size was made, whereby class 1–3 (high risk, frail patients) and class 4–6 (low risk, fit patients) were then grouped and used for further analysis.

The statistical significance level was set to 0.05. All computations were performed using STATA 16.1 (StataCorp. 2019. *Stata Statistical Software: Release 16*. College Station, TX: StataCorp LLC).

## Results

Overall, 548 patients treated for a CT scan confirmed FFP within the defined time period could be identified in the clinic information system. Eighty-six patients were treated operatively and thus excluded from this analysis. Among the remaining 462 patients, survival data could not be obtained from 34 patients. Hence, 428 patients were considered for this study. The median follow-up time was 59 months, and one year mortality was 18.5%. In total, 225 patients died in the course of follow-up.

Patient characteristics are depicted in Table 1. The mean age was 83.7 years (range 61–104 years), the majority were women (83.4%) and 2/3 still living in their home. Before the accident, two third were mobile without walking aid. Half of the patients had 3 or more relevant comorbidities. Most patients were discharged either to a rehabilitation centre or nursing home. In-hospital mortality was 0.7%.

**Table 1:** Patient characteristics

**Age at first hospital contact (*n*= 428)**	
Mean (10%-90% quantile)	83.7 (79.0–89.0)
< 80	116 (27.1%)
80–84	79 (18.5%)
85–89	135 (31.5%)
≥ 90	98 (22.9%)
**Gender (*n*= 428)**	
female	357 (83.4%)
male	71 (16.6%)
**Living situation at baseline (*n*= 325)**	
home alone	101 (31.1%)
home with partner	72 (22.2%)
home with support	44 (13.5%)
nursing home	108 (33.2%)
**Walking aid at baseline (*n*= 327)**	
with aid	201 (61.5%)
without aid	126 (38.5%)
**Parker mobility score (*n*= 312)**	7.2 (6.0–9.0)
1–5	57 (18.3%)
6	56 (17.9%)
7–8	36 (11.5%)
9	163 (52.2%)
**Elixhauser comorbidity score (*n*= 428)**	3.0 (2.0–4.0)
0	25 (5.8%)
1	79 (18.5%)
2	84 (19.6%)
3–4	148 (34.6%)
≥ 5	92 (21.5%)
**Place of accident (*n* = 413)**	
inside	310 (75.1%)
outside	103 (24.9%)
**Discharge to (*n*** **= 428)**	
home	34 (7.9%)
rehabilitation	329 (76.9%)
back to nursing home	58 (13.6%)
in-hospital mortality	3 (0.7%)
unknown	4 (0.9%)

The frequency of the observed specific fracture characteristics defined above and the FFP classification are depicted in Table 2.

**Table 2:** Frequency of fracture characteristics and the subgroups of the FFP classification

**Individual fracture characteristics (*n*= 428)**	
Extent of dorsal fractures	
0 - absent	89 (20.8%)
1 - unilateral	286 (66.8%)
2 - bilateral	53 (12.4%)
Horizontal sacral fracture	
absent	368 (86.0%)
present	60 (14.0%)
Extent of ventralfractures	
0 - absent	26 (6.1%)
1 - unilateral	359 (83.9%)
2 - bilateral	43 (10.0%)
Comminuted ventral fracture	
absent	365 (85.3%)
present	63 (14.7%)
Dislocated ventral fracture	
absent	319 (74.5%)
present	109 (25.5%)
**FFP classification (*n* = 428)**	
Ia	84 (19.6%)
Ib	7 (1.6%)
IIa	22 (5.1%)
IIb	84 (19.6%)
IIc	132 (30.8%)
IIIa	10 (2.3%)
IIIb	0 (0%)
IIIc	35 (8.2%)
IVa	0 (0%)
IVb	40 (9.3%)
IVc	14 (3.3%)

### Patient characteristics and mortality

**Fig. 2:**
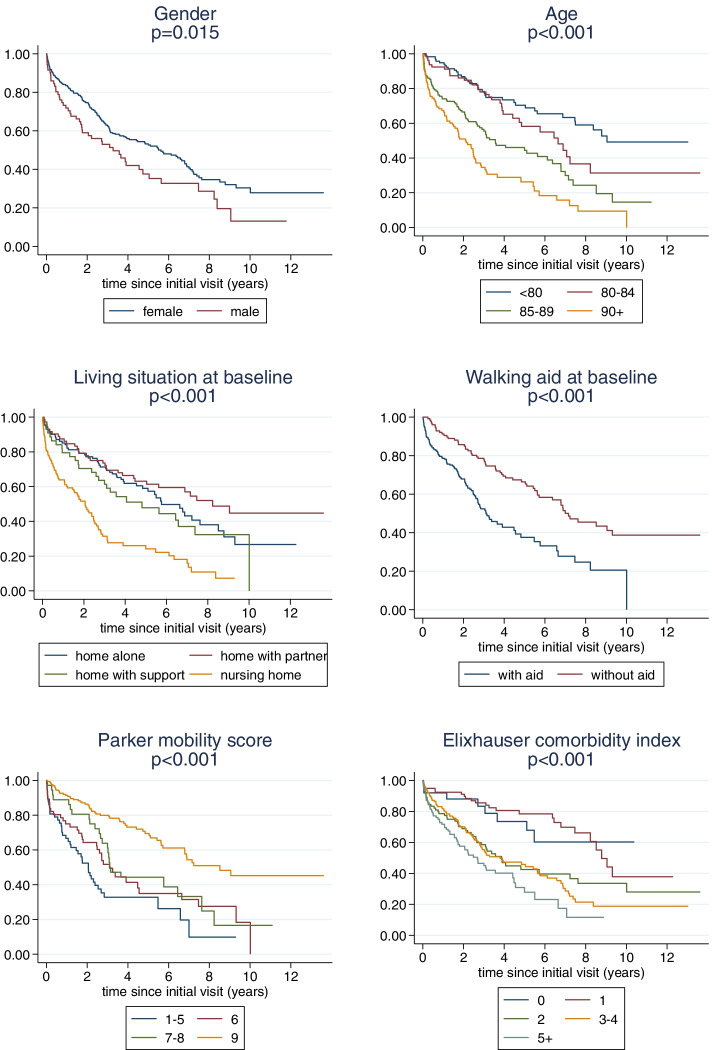
Relation of patient characteristics to mortality

The relation of the six analysed baseline patient characteristics to survival is shown in Fig. 2. We observe clear associations: male gender, higher age, living in a nursing home, use of walking aids, any deficit in mobility indicated by a PMS below the optimal value of 9, and two or more comorbidities are associated with poor survival in patients with a conservatively treated low-energy pelvic ring fracture. A multivariate analysis confirmed an independent prognostic value of gender (*p* = 0.001), age (*p* < 0.001) and comorbidities measured by ECI (*p* < 0.001) (Table 3). The significant effects of age, gender and comorbidity allowed us to conclude that these three variables carry an independent prognostic value. The other three variables did not show a significant effect, but they showed effects in the expected directions (worse survival if living at a nursing home or alone or if using walking aids, decreased survival with lower PMS).

**Table 3:** Multivariate analysis of patient characteristics and construction of risk index

	HR	*P*—value	95% CI
Gender (male vs female)	1.81	**0.001**	1.28–2.57
Age (in years)	1.06	** < 0.001**	1.04–1.08
Living situation		0.104	
home alone	Reference		
home with partner	0.88		0.56–1.38
home with support	0.73		0.44–1.20
nursing home	1.25		0.83–1.90
walking aid	0.71	0.133	0.45–1.11
PMS	0.95	0.294	0.86–1.05
ECI	1.24	** < 0.001**	1.14–1.36

### Fracture characteristics and mortality

**Fig. 3:**
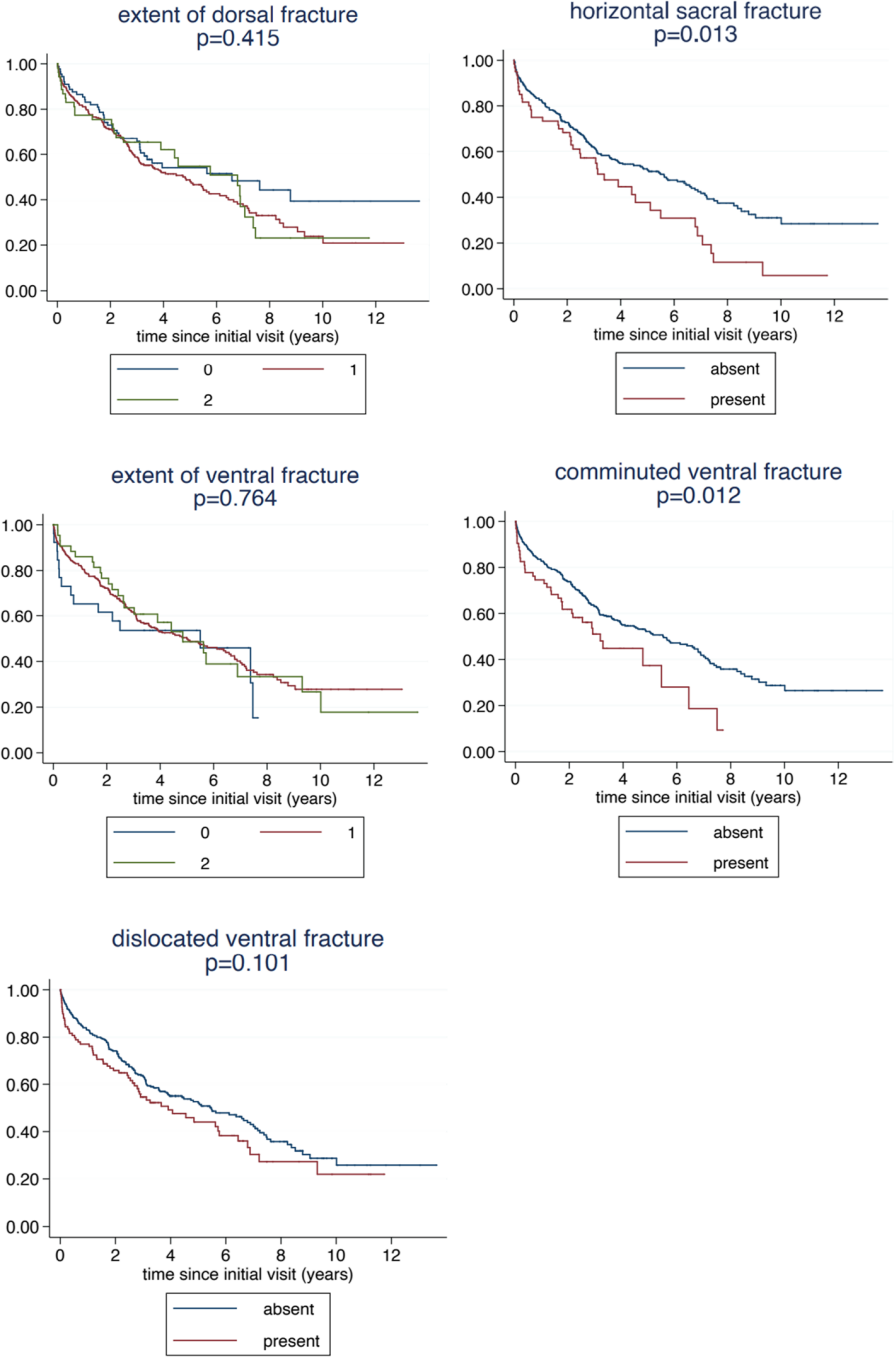
Relation of the individual fracture characteristics to survival

The correlation of the individual fracture characteristics with survival is shown in Fig. 3. We observe a significantly decreased survival in the presence of a horizontal sacral fracture and a comminuted ventral fracture. Survival was also decreased in patients with a dislocated ventral fracture, but this was not significant. For the extent of the fracture in the dorsal or ventral pelvic ring no association could be observed. A multivariate analysis confirmed an independent prognostic value of horizontal sacral and comminuted ventral fractures with hazard ratios (HR) of 1.55 (95% confidence interval (CI): 1.06, 2.27, *p* = 0.023) and 1.57 (95% CI: 1.07, 2.30, *p* = 0.020), respectively, indicating a relevant correlation with increased mortality (Table 4).

**Table 4:** Multivariate Analysis of fracture characteristics

	HR	*P-*value	95% CI
Extent of dorsal fracture	0.95	0.691	0.75–1.22
Horizontal sacral fracture	1.55	**0.023**	1.06–2.27
Extent of ventral fracture	0.91	0.570	0.65–1.27
Comminuted ventral fracture	1.57	**0.020**	1.07–2.30
Dislocated vent. fracture	1.17	0.327	0.85–1.60

### Fracture characteristics of frail and fit older patients and mortality

Further assessment of the relation of specific fracture characteristics separated for low-risk and high-risk patients was performed. Patients were grouped into 6 classes of equal size according to their overall mortality risk considering the following variables: age, gender, comorbidities, Parker mobility score, use of walking aids and living situation.

**Fig. 4:**
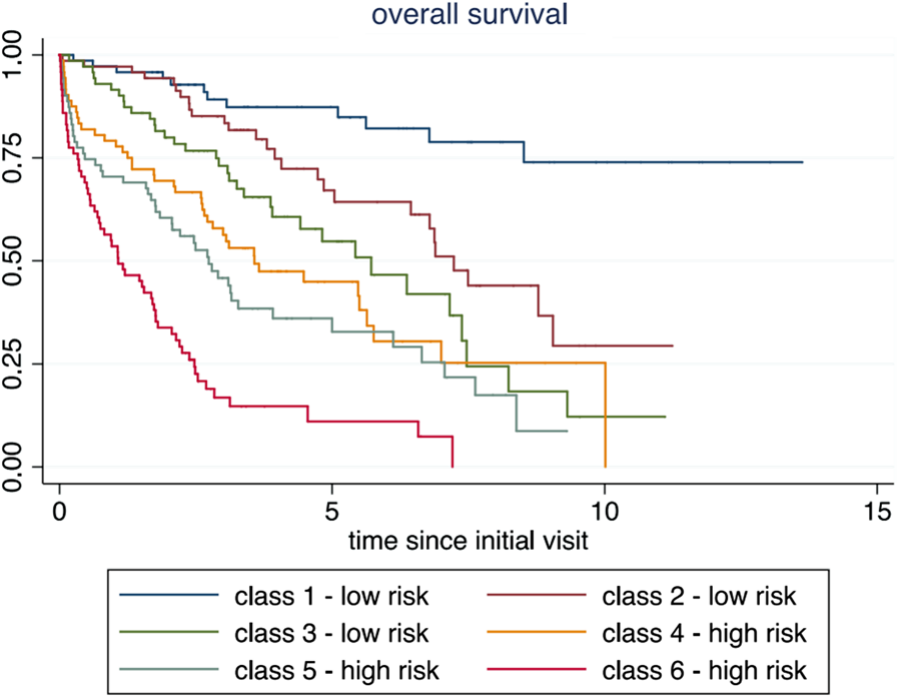
Survival according to stratified patients risk. The patients were grouped into six classes of equal size (see also Fig. 1)

Based on these findings, a risk index based on all six factors was constructed, allowing to differentiate clearly among low-risk and high-risk subjects (Fig. 1 and Fig. 4. In the following the terms “high-risk, frail patients” and “low-risk, fit patients” refer to the groups of patients in classes 1–3 and 4–6.

**Fig. 5:**
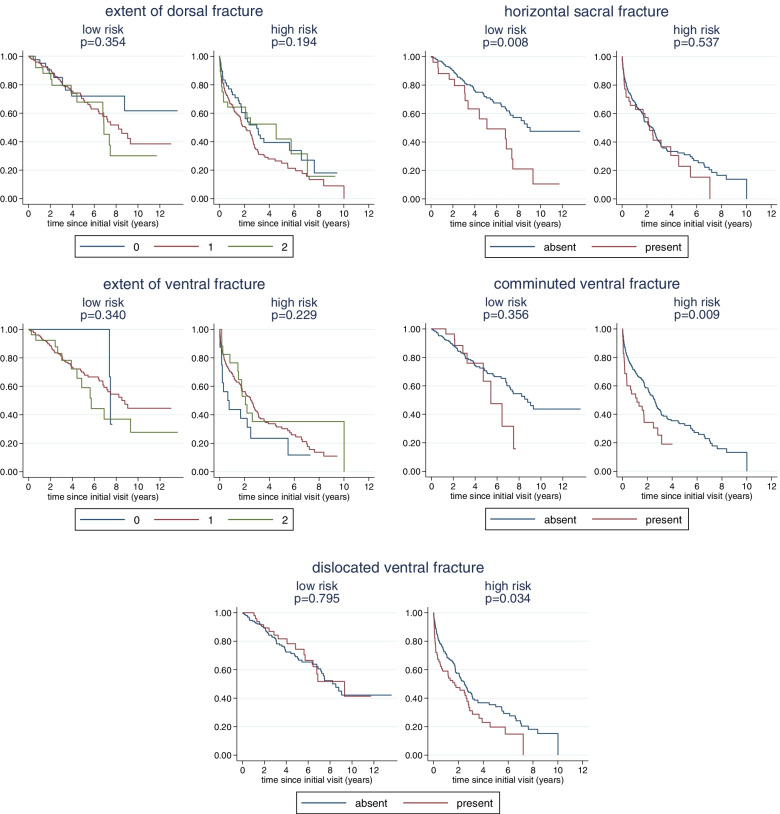
Relation of the individual fracture characteristics to survival within high- and low-risk patients

We observe a more pronounced association for the horizontal sacral fracture component in low-risk patients than in high-risk patients and a more pronounced association for the comminuted and dislocated ventral fractures in high-risk patients (Fig. 5). Separate multivariate analyses in high-risk and low-risk patients could confirm an independent prognostic value of horizontal sacral fractures in low-risk patients (HR = 2.23, 95% CI: 1.20, 4.15, *p* = 0.012) and the prognostic value of comminuted ventral fractures (HR = 1.71, 95% CI: 1.08, 2.68, *p* = 0.021) and dislocated ventral fractures (HR = 1.47, 95% CI: 1.01, 2.15, *p* = 0.044) in high-risk patients (Table 5). Comminuted ventral fractures still showed a HR of 1.55 in low-risk patients, but this did not reach significance.

**Table 5:** Multivariate analysis of fracture characteristics in low-risk and high-risk patients

**low risk patients**			
	HR	*P-*value	95% CI
Extent of dorsal fracture	1.18	0.426	0.78–1.77
Horizontal sacral fracture	2.23	**0.012**	1.20–4.15
Extent of ventral fracture	1.60	0.062	0.98–2.62
Comminuted ventral fracture	1.55	0.242	0.74–3.22
Dislocated ventral fracture	0.68	0.211	0.37–1.24
**high risk patients**			
	HR	*P-*value	95% CI
Extent of dorsal fracture	0.92	0.612	0.68–1.25
Horizontal sacral fracture	1.08	0.754	0.66–1.78
Extent of ventral fracture	0.63	0.054	0.39–1.01
Comminuted ventral fracture	1.71	**0.021**	1.08–2.68
Dislocated ventral fracture	1.47	**0.044**	1.01–2.15

### FFP and mortality

**Fig. 6:**
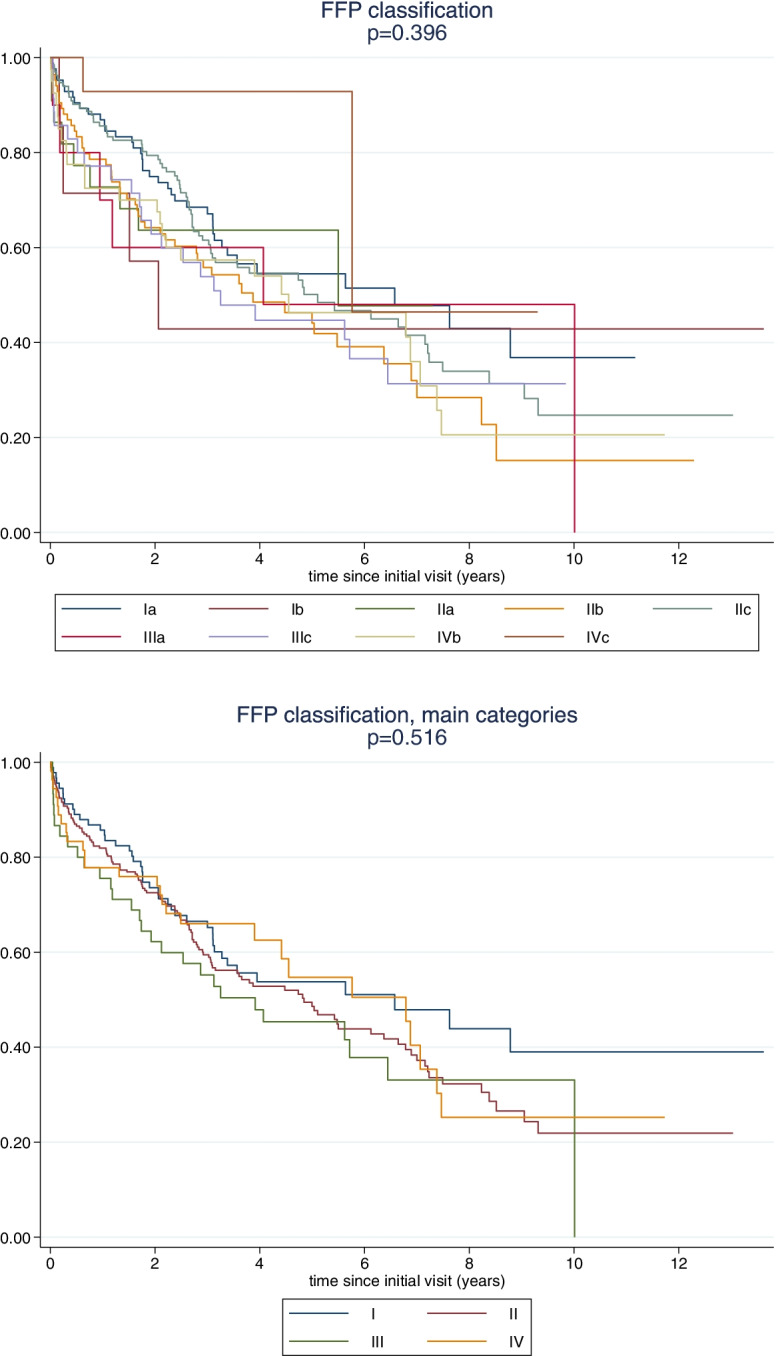
Correlation of the FFP classification to survival

No association of the FFP classification with survival was observed in this cohort of conservatively treated patients (Fig. 6). Neither the full classification with its 11 subcategories nor the concentration on the four main categories of the FFP classification showed a significant association with mortality.

## Discussion

In this study of a cohort of conservatively treated patients with a low-energy pelvic ring fracture, we could demonstrate a relation of three specific fracture characteristics, namely horizontal sacral fractures, comminuted ventral fractures and dislocated ventral fracture, with overall survival. In contrast, the extent of the fracture (absent, unilateral, bilateral) in the dorsal or ventral ring was not associated with overall survival.

Previously a significant difference in survival of older patients with an AO type A compared to an AO type B pelvic fractures after low-energy trauma with 80% of conservative treatment has been reported [20]. In contrast, the categories of the FFP classification have neither in our population nor in a previous study been reported to be associated with survival or mortality [21]. Also, the alphanumeric classification by Krappinger et al. has not yet been reported to correlate to outcome measures [25].

Our results suggest that specific aspects of the dorsal as well as ventral fracture seem to have an impact on survival and it might therefore be favourable to take these into account in future decision-making regarding treatment options for these patients. These three specific aspects of pelvic fractures in older patients seem to have a higher impact than the extent of dorsal or ventral fractures. Horizontal sacral fractures are most frequently associated with an H-type fracture pattern that often triggers surgical stabilisation, so the unfavourable prognosis associated with horizontal sacral fractures may by already taken into account. In contrast, the impact of comminuted and dislocated anterior fractures on mortality might reflect a so far underestimated functional impact of fractures in the ventral pelvic ring.

A stratification according to patients’ risk reveals further insight. Horizontal sacral fractures were found to be particularly harmful in low-risk patients, whereas comminuted and dislocated ventral fractures proved more harmful in high-risk patients with a significant increase in mortality in these patients. This impact of the dorsal vs anterior fracture characteristic in distinctly different populations of older patients is somewhat surprising. In general, high-risk patients are characterized in our population by a substantial short-term mortality (25% mortality within the first six months), whereas low risk patients are characterized by a slow long-term decline. As visible in Fig. 5, in frail, high risk patients, comminuted or dislocated ventral fractures seem to increase the short-term mortality, whereas horizontal sacral fracture do not show this effect. In contrast, in fit, low risk patients, horizontal sacral fractures seem to have an unfavourable effect on the long-term decline. These findings suggest that interpretation of fracture characteristics with respect to decision making may have to take into account the degree of frailty of the patients.

In line with this interpretation the impact of fracture characteristics on mortality seems to be smaller compared to patient characteristics. Age, existing decreased mobility, and comorbidities are directly associated with an increased mortality risk. The same tendency was observed for patient characteristics that did not directly and statistically relevantly correlate with mortality, ie. living situation, use of walking aid and PMS. The lack of significance can be explained by the fact that these three all tend to measure the same concept, namely the degree of mobility and as consequence independence in daily living. Indeed, if added as single variables to a base model with age, gender and comorbidity as covariates, they always resulted in a statistically significant effect. These findings underline the frailty of this specific patient group and the vital importance of mobility [26].

Treatment strategies for pelvic fragility fractures are not yet well established [2, 24, 27]. Results for relevant outcomes have been reported for patients treated either conservatively or operatively, but are still somewhat ambiguous with improved survival for operatively treated patients [19, 28]. In contrast, others reported an early protective effect of the non-operative treatment [29]. One-year mortality after surgical fracture stabilisation has been reported between 10–28% [21, 30].

We reported only on conservatively treated patients. The restriction of this analysis to conservatively treated patients avoids an undesirable effect of surgery as a confounding factor. Surgery can unpredictably impact outcome and survival in both directions with a potential improvement, but also with a potential deterioration due to postoperative complications and surgery-related morbidity. Operatively treated patients with a pelvic fragility fracture might represent a selected subpopulation. However, even if the decision for surgery depends directly on the fracture characteristics, this does not bias the estimation of the effect of the prognostic value of these factors [31].

In addition, the analysis of conservatively treated patients allows to discuss possible adaptations of current practice and specific analyses of potentially underserved subpopulations.

The majority of our population was independently mobile and living prior to the diagnosis of a pelvic ring fracture. Almost a quarter of this conservatively treated population further presented with a FFP type III or IV. Furthermore, treatment strategies generally aim at restoring baseline mobility and independence, thus first evaluating the success of conservative treatment. If conservative treatment was not successful, that is, if mobilisation was associated with persistent pain, we have considered and discussed with the patient the recommendation of a surgical stabilisation of the fracture. An assessment of the fracture morphology, but also of the patient's baseline performance, risk factors and outcome expectations played thereby an important role.

We acknowledge the limitations of our study. First, it is a retrospective analysis. As follow up information was restricted to survival, data could nevertheless reliably be obtained for almost all patients using official records. Nevertheless, using overall survival as measure probably allows only to have a look at the tip of the iceberg with the true triggers and points of possible interventions hidden. Rigorous follow-up assessments with respect to mobility and quality of life may produce better insights into the association with patient relevant outcomes.

## Conclusion

Specific fracture characteristics such as horizontal sacral fracture, comminuted and dislocated ventral fractures may indicate a higher mortality risk of patients with a low-energy pelvic ring fracture. Hence, these fracture characteristics should be taken into account in future treatment algorithms and decisions on patient management.

## Data Availability

The dataset generated and analysed during the current study are not publicly available due to confidentiality but are available from the corresponding author on reasonable request.
